# Emergency medicine matters: epidemiology of medical pathology and changes in patient outcomes after implementation of a post-graduate training program at a Tertiary Teaching Hospital in Kigali, Rwanda

**DOI:** 10.1186/s12245-021-00331-2

**Published:** 2021-01-21

**Authors:** Katelyn Moretti, Doris Lorette Uwamahoro, Sonya Naganathan, Chantal Uwamahoro, Naz Karim, Menales Nkeshimana, Adam R. Aluisio

**Affiliations:** 1 Department of Emergency Medicine, Warren Alpert Medical School, Brown University, Providence, USA; 2 Department of Anesthesia, Emergency Medicine and Critical Care, University of Rwanda, Kigali, Rwanda

**Keywords:** Medical pathology, Low-middle income countries, Emergency medicine training, Residency, Rwanda

## Abstract

**Background:**

Emergency care is a new but growing specialty across Africa where medical conditions have been estimated to account for 92% of all disability-adjusted life years. This study describes the epidemiology of medical emergencies and the impact of formalized emergency care training on patient outcomes for medical conditions in Rwanda.

**Methods:**

A retrospective cohort study was performed using a database of randomly sampled patients presenting to the emergency center (EC) at the University Teaching Hospital of Kigali. All patients, > 15 years of age treated for medical emergencies pre- and post-implementation of an Emergency Medicine (EM) residency training program were eligible for inclusion. Patient characteristics and final diagnosis were described by time period (January 2013–September 2013 versus September 2015–June 2016). Univariate chi-squared analysis was performed for diagnoses, EC interventions, and all cause EC and inpatient mortality stratified by time period.

**Results:**

A random sample of 1704 met inclusion with 929 patients in the pre-residency time period and 775 patients in the post-implementation period. Demographics, triage vital signs, and shock index were not different between time periods. Most frequent diagnoses included gastrointestinal, infectious disease, and neurologic pathology. Differences by time period in EC management included antibiotic use (37.2% vs. 42.2%, *p* = 0.04), vasopressor use (1.9% vs. 0.5%, *p* = 0.01), IV crystalloid fluid (IVF) use (55.5% vs. 47.6%, *p* = 0.001) and mean IVF administration (2057 ml vs. 2526 ml, *p* < 0.001). EC specific mortality fell from 10.0 to 1.4% (*p* < 0.0001) across time periods.

**Conclusions:**

Mortality rates fell across top medical diagnoses after implementation of an EM residency program. Changes in resuscitation care may explain, in part, this mortality decrease. This study demonstrates that committing to emergency care can potentially have large effects on reducing mortality.

## Introduction

Health initiatives worldwide have historically focused on primary preventative strategies and communicable disease specific interventions [1]. However, in 2019, The World Health Organization specifically recognized the importance of a functional emergency care system and named it “an essential component of quality care, and that millions of deaths and long-term disabilities could be prevented if emergency care services exist and patients reach them in time.” [2]. Even so, a systemic approach to emergency medicine (EM) is often missing despite demonstrations of its necessity [3]. Often, clear data from low- and middle-income countries (LMICs) on the impact of quality emergency care is relegated to a specific disease states and the outcomes from systemic integration of emergency care into the healthcare system is overlooked [4].

While traumatic injuries are often the focus when discussing the need for emergency care, LMICs bear a disproportionate burden of non-traumatic pathology including infectious disease, maternal complications and malnutrition [5]. In addition, as the prevalence of non-communicable diseases (NCDs) increase, their proportional mortality rates are also increasing [6]. In 2011, a consensus conference was held with sub-Saharan African leaders during which acute care was identified as necessary and underdeveloped [7]. One barrier highlighted was the underreporting of the burden of acute diseases; medical conditions have been estimated to account for 92% of all disability-adjusted life years across Africa [3].

Rwanda is a LMIC which has identified emergency medicine as a key priority [8]. The first EM residency training program in the country was started at the University Teaching Hospital of Kigali (CHUK) in August 2015. Since the implementation of the emergency care system at CHUK, overall hospital mortality likelihood has fallen by 43% [9]. While the epidemiology of traumatic pathology has been previously described [10, 11], the specific burden from non-traumatic diseases and the impact of the implementation of a system of emergency care on this population remains unknown. In the following analyses, patient characteristics and diagnoses, EM interventions, and patient mortality are quantified and described across pre- and post-residency implementation time periods.

## Methods

This retrospective cohort study was performed using a previously established database out of the University Teaching Hospital of Kigali (CHUK) in Kigali, Rwanda [12]. This hospital represents the primary national public referral center of Rwanda and is an urban, tertiary-care institution with approximately 40 emergency beds and 500 inpatient beds. In August 2015, a 4-year EM residency training program was launched as previously described [8]. This resulted in a model of EM resident staffing with specialist oversight. Additional changes included point-of-care ultrasound, formalized triage, team-based resuscitation, and the implementation of patient acuity zones. No changes were made to resource allocation nor to the physical structure of the department. EMS advice provided by ED physicians may also have been affected by this model, with terminal patients remaining at home or the deceased pronounced in the prehospital setting.

Patients 16 years of age or older presenting to the EC during the time periods of January–September 2013 and September 2015–June 2016 with a primary medical complaint were eligible for inclusion. Data was collected in an interrupted time series to evaluate the effects of implementation of this new residency program [10].

Cases were identified from the CHUK Emergency Database whose creation has been previously described [9, 11–14]. In summary, all EC cases during the study period were identified and linked to corresponding medical records via a multipoint composite index and a random sample of cases were accrued based on month of EC visitation for analysis. Data was then extracted from medical records using a standardized instrument.

### Data management

All patients with an initial triage categorization of medical, age greater than or equal to 15 with a final diagnosis recorded were included and stratified based on time period. Patients were categorized as medical vs. trauma based on clinical data provided from the source charts in which the South Africa Triage Scale was used. The South Africa Triage Scale categorizes patients as trauma vs. non-trauma. Those classified as non-trauma, were coded as medical [15].

Patient characteristics including gender, age, triage vital signs, and shock index were described by time period. Primary final diagnosis was categorized by physiologic system. Inpatient and EC deaths were aggregated for overall mortality outcomes. Patients who eloped or were transferred to a different institution were analyzed as survivals.

Subgroup analyses by final diagnosis examined inpatient and EC mortality pre- and post-residency implementation. Selected EC interventions of interest included intravenous (IV) crystalloid administration, blood product transfusion, antibiotic administration, vasopressor administration, use of supplemental oxygen, and intubation and were analyzed as binary (given/not given). All forms of vasopressors aggregated for analysis. IV crystalloids included lactated ringers and normal saline administration. Mean volume of IV crystalloid fluid administration was analyzed among those receiving fluids.

Supplemental oxygen included the following non-invasive methods: nasal cannula, face mask or non-rebreather mask. Intubation was analyzed as binary—performed and not performed. Triage vital signs are reported as median with interquartile ranges (IQR). Shock index (heart rate divided by systolic blood pressure) was calculated and dichotomized by ≤ 0.9 and > 0.9 based on previous studies [16, 17].

### Data analysis

Data analysis was performed using STATA version 15.0 (StataCorp.; College Station, USA). Descriptive analysis was performed for the overall cohort stratified by the time period. Variables were described using frequencies with percentages. Final diagnoses were categorized described using frequencies with percentages. Univariate chi-squared analysis was performed comparing pre- and post-residency implementation for all-cause, EC, and in-patient mortality overall and for each of the top 6 final diagnosis categories by time period. Univariate chi-squared analysis was performed for EC interventions for each of the top 6 final diagnoses by time period.

## Results

**Fig. 1:**
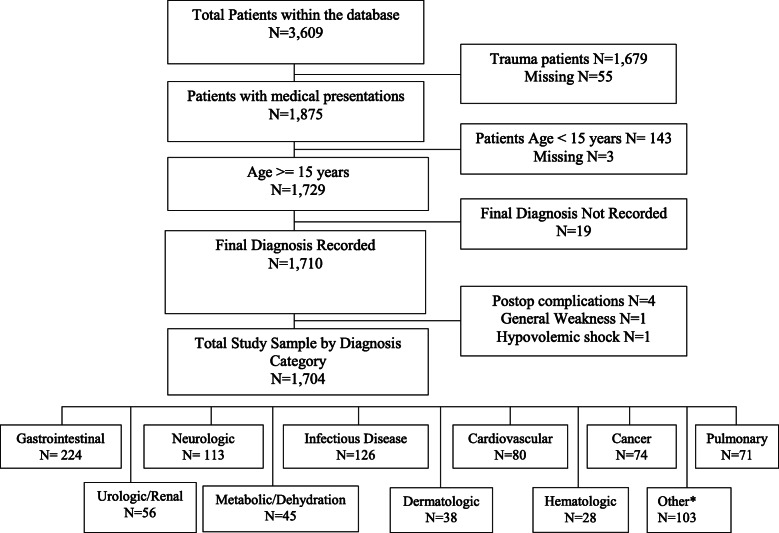
Study sample. *Other includes: ENT, Obstetrics, Endocrinology, Poisoning, Ophthalmology, Psychopathologies

A study sample of 1704 met inclusion criteria. There were 929 cases in the pre-residency time period (January–September 2013) and 775 cases in the post-residency implementation time period (September 2015–June 2016). Of the initial cohort, 83 cases (2.2%) were excluded for missing data (Fig. 1).

Patient characteristics including gender, age distribution, triage vital signs, and shock index were similar across both time periods (Table 1). In both cohorts, there was a slight male predominance (52.9 and 53.9%) and the majority of patient presented between the ages of 16 to 44 years (55.4 and 57.6%) with an equal proportion of shock index greater than 0.9 (29.0 and 27.7%).

**Table 1:** Patient presenting characteristics, triage vital signs, and shock index by time period (January–September 2013 and September 2015–June 2016)

	Pre-residency (***N*** = 929)	Post-residency implementation (***N*** = 775)
**Sex (%)**		
Male	491 (52.9)	418 (53.9)
Female	438 (47.2)	357 (46.1)
Missing	0 (0)	0 (0)
**Age (%)**		
Overall median (IQR)	41 (29, 59)	40 (28, 60)
16–44	515 (55.4)	446 ( 57.6)
45–65	255 (27.5)	182 (23.5)
> = 65	159 (17.1)	147 (19.0)
Missing	0 (0)	0 (0)
**Vital signs, median (IQR)**		
Heart rate	98 (83, 115)	96 (81, 112)
Respiratory rate	20 (20, 22)	20 (18, 20)
Systolic blood pressure	123 (107, 141)	119 (106, 135)
**Shock index (%)**		
</= 0.9	466 (50.2)	388 (50.1)
> 0.9	269 (29.0)	215 (27.7)
Missing	194 (20.0)	172 (22.2)

**Fig. 2:**
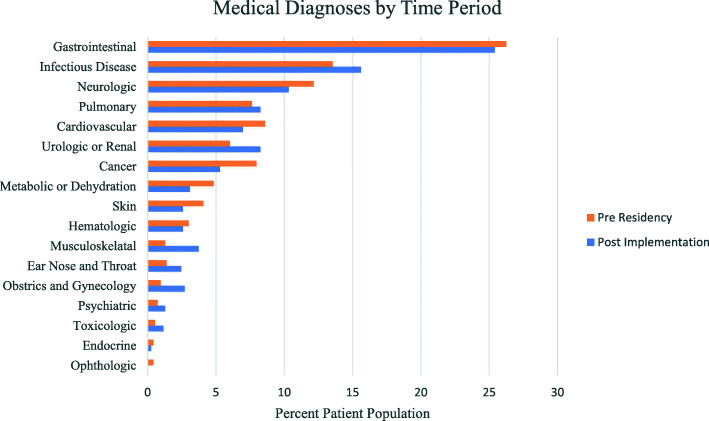
Percent patient population with medical diagnoses by physiologic system stratified by time period

Prevalence of the top 6 systems causing pathology in medical patients was not different between time periods (Fig. 2) and included in decreasing prevalence: gastrointestinal (26.3% vs. 25.4%, *p* = 0.69), infectious disease (13.6% vs. 15.6%, *p* = 0.23), neurologic (12.2% vs. 10.3%, *p* = 0.23), pulmonary (7.6% vs. 8.3%, *p* = 0.64), cardiovascular (8.6% vs. 7.0%, *p* = 0.21), and urologic or renal (6.0% vs. 8.3%, *p* = 0.07). These six categories encompassed 75% of the total patient cohort.

Differences in EC management observed across the time periods includes increased use of antibiotics (37.2% vs. 42.2%, *p* = 0.04) and decreased use of vasopressors (1.9% vs. 0.5%, *p* = 0.01). A decrease in percent patients receiving IV crystalloid fluid resuscitation was noted (55.5% vs. 47.6%, *p* = 0.001). However, mean amount of crystalloid fluids received when administered increased across time periods (2057 ml vs. 2526 ml, *t* test = 0.0001, Table 2).Overall facilities based all-cause patient mortality decreased between time periods (19.7% vs. 14.0%, *p* = 0.002). There was no statistical difference in inpatient mortality. ED specific mortality fell from 10.0% to 1.4% (*p* < 0.0001) with statistically decreased ED mortality for patients diagnosed with gastrointestinal, infectious, neurologic, and cardiovascular disease (Table 3).

**Table 2:** EC interventions stratified by time period and top 6 diagnoses

EC intervention	Pre-residency	Post-residency	Chi^**2**^
**Overall**	***N*** **= 937**	***N*** **= 780**	
IV crystalloid infusion	516 (55.5)	369 (47.6)	0.001
IV fluid amount (ml)	2057	2526	*t* test = 0.0001
Blood products transfused	88 (16.1)	58 (14.8)	0.6
Vasopressors	18 (1.9)	4 (0.5)	0.01
Intubation	29 (3.1)	13 (1.7)	0.06
Antibiotics	346 (37.2)	327 (42.2)	0.04
O2 therapy	169 (18.2)	113 (14.6)	0.046
**Gastrointestinal**	***N*** **= 244**	***N*** **= 197**	
IV crystalloid infusion	169 (69.3)	125 (63.5)	0.2
IV fluid amount (ml)	2784.5	3266.8	*t* test = 0.04
Blood products transfused	20 (8.2)	12 (6.1)	0.4
Vasopressors	4 (1.6)	1 (0.5)	0.3
Intubation	3 (1.2)	2 (1.0)	0.8
Antibiotics	100 (40.9)	100 (50.7)	0.04
O2 therapy	20 (8.2)	12 (6.1)	0.4
**Infectious disease**	***N*** **= 126**	***N*** **= 121**	
IV crystalloid infusion	79 (62.7)	62 (51.2)	0.07
IV fluid amount (ml)	2033.8	2250	*t* test = 0.5
Blood products transfused	12 (9.5)	8 (6.6)	0.4
Vasopressors	3 (2.4)	0 (0)	0.09
Intubation	4 (3.2)	5 (4.1)	0.7
Antibiotics	75 (59.5)	74 (61.2)	0.8
O2 therapy	35 (27.8)	16 (13.2)	0.005
**Neurologic**	***N*** **= 113**	***N*** **= 80**	
IV crystalloid infusion	62 (54.9)	37 (46.3)	0.4
IV fluid amount (ml)	1416	1595	*t* test = 0.5
Blood products transfused	3 (2.7)	0 (0)	0.2
Vasopressors	1 (0.9)	1 (1.25)	0.8
Intubation	13 (11.5)	2 (2.5)	0.02
Antibiotics	35 (31.0)	26 (32.5)	0.8
O2 therapy	29 (25.7)	13 (16.3)	0.1
**Pulmonary**	***N*** **= 71**	***N*** **= 64**	
IV crystalloid infusion	29 (40.9)	27 (42.2)	0.9
IV fluid amount (ml)	1574	1761	*t* test = 0.5
Blood products transfused	1 (1.4)	2 (3.1)	0.5
Vasopressors	0 (0)	0 (0)	
Intubation	2 (2.8)	1 (1.6)	0.6
Antibiotics	24 (33.8)	30 (46.9)	0.1
O2 therapy	27 (38.0)	28 (43.8)	0.5
**Cardiovascular**	***N*** **= 80**	***N*** **= 54**	
IV crystalloid infusion	32 (40.0)	10 (18.5)	0.009
IV fluid amount	1629	2700	*t* test = 0.2
Blood products transfused	5 (6.2)	4 (7.4)	0.79
Vasopressors	6 (0.4)	2 (3.7)	0.4
Intubation	2 (2.5)	1 (1.9)	0.8
Antibiotics	18 (22.5)	9 (16.7)	0.4
O2 therapy	30 (37.5)	19 (35.2)	0.8
**Urologic or renal**	***N*** **= 56**	***N*** **= 64**	
IV crystalloid infusion	18 (32.1)	15 (23.4)	0.29
IV fluid amount	1233	1844	*t* test = 0.17
Blood products transfused	4 (7.1)	2 (3.1)	0.31
Vasopressors	2 (3.6)	0 (0)	0.13
Intubation	1 (1.8)	0 (0)	0.28
Antibiotics	11 (19.6)	18 (28.1)	0.28
O2 therapy	5 (8.9)	9 (14.1)	0.38

**Table 3:** All-cause, emergency center, and inpatient mortality stratified by time period

All-cause mortality	Pre-residency (***N*** = 929)	Post-residency implementation (***N*** = 775)	Chi^**2**^
Overall	170 (19.9)	97 (14.0)	0.002
Gastrointestinal	29 (12.8)	18 (9.9)	0.4
Infectious disease	37 (30.0)	23 (22.1)	0.2
Neurologic	24 (24.0)	12 (12.1)	0.3
Cardiovascular	18 (25.4)	9 (18.4)	0.4
Cancer	23 (32.4)	17 (42.5)	0.3
Pulmonary	14 (22.6)	7 (13.2)	0.2
**EC mortality**			
Overall	86 (10.0)	10 (1.4)	< 0.0001
Gastrointestinal	14 (6.2)	1 (0.6)	0.003
Infectious disease	19 (15.5)	5 (4.8)	0.009
Neurologic	15 (14.9)	0 (0)	0.001
Cardiovascular	12 (16.9)	1 (2.0)	0.009
Cancer	5 (8.1)	1 (1.9)	0.1
Pulmonary	5 (6.9)	2 (5.0)	0.7
**Inpatient mortality**^**a**^	***N*** **= 522**	***N*** **= 539**	
Overall	84 (16.1)	87 (16.1)	1.0
Gastrointestinal	15 (10.9)	17 (12.0)	0.8
Infectious disease	18 (22.5)	18 (24.3)	0.8
Neurologic	9 (15.3)	12 (21.1)	0.4
Pulmonary	9 (36.0)	6 (16.7)	0.1
Cardiovascular	6 (14.6)	8 (18.2)	0.7
Urologic or renal	3 (9.1)	5 (12.5)	0.6

^a^Incidence of inpatient mortality calculated from amongst those admitted

When ED interventions were analyzed for each of the top 6 diagnosis categories by time period, trends in increasing frequency of antibiotic administration were noted for cases with infectious diseases, neurologic pathology and cancer. However, statistically significant increases were seen only for gastrointestinal pathology (40.9% vs. 50.7%, *p* = 0.04). A decrease in vasopressor use was seen for gastrointestinal pathology, infectious disease, cardiovascular disease, and cancer, although none were statistically significant. Although only cardiovascular reached statistical significance, a trend toward decreasing frequency of IV fluid administration was noted across the top 5 diagnoses, increasing only in the sixth diagnosis of pulmonary pathology. The mean amount administered increased across all six diagnoses, although only gastrointestinal pathology reached statistical significance.

## Discussion

The top 6 physiologic systems of medical pathologies across both time periods found in the emergency department were gastrointestinal, infectious disease, neurologic, pulmonary, cardiovascular, and urologic or renal. Changes in EC interventions and mortality rates across time periods demonstrate the potential impacts EC care can have on patients with medical pathology. Understanding the overall medical burden of disease allows for targeted disease analyses and interventions that will result in the greatest impact.

Previous studies examining the burdens of medical pathology amongst EC patients in Africa are limited, with just one additional study identified examining all patients (including pediatrics) presenting to the EC. In a study from Ifakara, Tanzania, infectious disease and trauma were noted as primary diagnoses. However, similar to cardiovascular rates seen in the present data, hypertensive emergency was noted as the leading diagnosis in those > 50 years of age [18]. Disease-specific studies in Tanzania have noted similarly high rates of cardiovascular and neurologic pathology [19] as well as renal disease [20] and gastrointestinal pathology [21] as seen in this study. Thus, this study helps to establish a foundation of the epidemiology of medical pathology amongst adult patients in LMICs and more specifically, Africa which will facilitate better understanding of care and resource needs in those settings. In addition, the consistency noted with data from Tanzania suggests external validity of results reported here.

After the implementation of an emergency medicine residency, a decrease in overall medical mortality, driven by EC mortality, was seen. The lack of change in inpatient mortality rates suggest these findings are, in fact, driven predominately by impacts during early care delivery in the EC setting. In sub-analyses, EC mortality decreased in the top 5 diagnosis categories. Similar findings of overall decreased mortality rates were seen after institution of an emergency medicine residency program in Tanzania although specific changes in EC management were not described [21].

Specific EC interventions were analyzed across time periods and by systems in order to attempt to assess for management changes that could have contributed to observed decreased mortality. After the initiation of residency training, a more targeted and aggressive use of IV fluids. While fewer patients received intravenous fluids, those that did received a larger amount. An assumed subsequent decrease in the use of vasopressors, oxygen therapy (for neurologic patients), and intubations (for neurologic patients) was found. One potential explanation for these results includes fewer patient complications from IVF administration as the subset of fluid responsive patients were more adequately identified. This trend was maintained in gastrointestinal, infectious disease, neurologic, cardiovascular, and urologic/renal pathologies. However, given small sample sizes within sub-analyses, statistical significance was reached only for increased mean amount IVF for gastrointestinal patients and decreased frequency of IVF for cardiovascular patients. These findings may suggest improved, targeted resuscitation is contributing to improved survival.

In addition, overall use of antibiotics increased in the post-residency time period. This trend was maintained across gastrointestinal, infectious disease, neurologic, pulmonary, and renal although statistical significance was reached only in the first two categories. A decrease was seen in cardiovascular pathologies. When looking at more specific diagnoses within each category (Table 4), all groups with trends in antibiotic increase had infectious causes of top diagnoses. Only cardiovascular, where a decrease in antibiotic use was seen, did not have an infectious cause of a top specific diagnosis. However, the data do not have sufficient detail to fully explore the utilization of all treatments across all medical pathologies and conclusions on causation cannot be drawn.

**Table 4:** Top 5 specific diagnoses within the top 6 diagnosis categories.

**Gastrointestinal**	**Infectious disease**	**Neurologic**
Intestinal obstruction (20.7%)	Malaria (29.8%)	Stroke (ischemic or hemorrhagic) (32.9%)
Gastritis/gastric ulcer (13.4%)	Tuberculosis (27.7%)	Meningitis/encephalitis (15.1%)
Acute abdomen/peritonitis (12.3%)	HIV/AIDS-related illness (20.8%)	Headache (13.5%)
Hernia (8.2%)	Sepsis (unknown source) (9.4%)	Seizure (7.8%)
Cirrhosis (5.5%)	Other (3.8%)	Other (7.3%)
**Pulmonary**	**Cardiovascular**	**Urologic/renal**
Asthma/COPD (41.5%)	Acute heart failure (34.2%)	Chronic kidney failure with acute exacerbation (21.7%)
Pneumonia (28.9%)	Hypertension/hypertensive emergency (33.3%)	Urinary tract infection (20.0%)
Pulmonary effusion (8.9%)	Dilated cardiomyopathy (10.3%)	Acute kidney injury (10.0%)
Pulmonary embolism (5.2%)	Valvular disease (5.1%)	Benign prostatic hypertrophy (7.5%)
Other (5.2%)	Other (4.3%)	Other (7.5%)

Given that there was no change in inpatient mortality across time periods, inpatient interventions were not analyzed.

## Limitations

Given the retrospective nature of the chart review, a proportion of cases were deemed ineligible due to missing data which may introduce bias. However, records are assumed to be missing at random and should therefore represent limited bias. In addition, the retrospective nature of the dataset does not allow for analysis of causality.

While illness severity was evaluated with triage vital signs and shock index, the dynamics of patient acuity were not captured, and confounding is possible. In addition, while categories of patient diagnoses were identified, specific patient pathologies were not examined in relationship to changing management given small observed numbers. Future studies will further evaluate specific diagnoses within leading categories of non-traumatic pathology.

Given the nature of the data, only specific interventions and outcomes were able to be analyzed. Other implementations during the residency such as a triage system, bedside point of care ultrasound and procedural training may be contributing to mortality decreases post-residency implementation. Additional cofounders may also be present. During this time, the Ministry of Health supported similar program development within internal medicine, surgery and anesthesia. Thus, time to consultant intervention and consultant practices may have changed [22]. While mortality rates fell in Rwandan Hospitals during the time of this study, the magnitude of change was greater within this cohort suggesting interventions intrinsic to the department outlined above were contributing to the observed outcomes [23].

Medical disease accounts for the majority of pathology across Africa. However, it remains poorly described. Understanding the distribution of medical pathology by physiologic system allows for more impactful patient-centered research. In addition, while emergency medicine residency training is now accepted in high-income countries, the emergency medicine specialty remains rare or non-existent in LMIC [24]. However, in this study, a formalized emergency medicine program appears to have improved medical patient outcomes, potentially from targeted, aggressive fluid resuscitation. Results reported here add to the growing compendium of data demonstrating that emergency medicine and acute care should be viewed as a necessity, not a luxury.

## Data Availability

The datasets generated and analyzed during the current study are not publicly available due to patient confidentiality but are available from the corresponding author on reasonable request.
